# Information Search and Financial Markets under COVID-19

**DOI:** 10.3390/e22070791

**Published:** 2020-07-20

**Authors:** Behzod B. Ahundjanov, Sherzod B. Akhundjanov, Botir B. Okhunjanov

**Affiliations:** 1 Department of Economics, Bucknell University, 1 Dent Drive, Lewisburg, PA 17837, USA; ba019@bucknell.edu; 2 Department of Applied Economics, Utah State University, 4835 Old Main Hill, Logan, UT 84322-4835, USA; 3 School of Economic Sciences, Washington State University, Hulbert Hall 101, Pullman, WA 99164-6210, USA; botir.okhunjanov@wsu.edu

**Keywords:** COVID-19, information, Google trends, risk perception, financial markets, SVAR

## Abstract

The discovery and sudden spread of the novel coronavirus (COVID-19) exposed individuals to a great uncertainty about the potential health and economic ramifications of the virus, which triggered a surge in demand for information about COVID-19. To understand financial market implications of individuals’ behavior upon such uncertainty, we explore the relationship between Google search queries related to COVID-19—information search that reflects one’s level of concern or risk perception—and the performance of major financial indices. The empirical analysis based on the Bayesian inference of a structural vector autoregressive model shows that one unit increase in the popularity of COVID-19-related global search queries, after controlling for COVID-19 cases, results in 0.038–0.069% of a cumulative decline in global financial indices after one day and 0.054–0.150% of a cumulative decline after one week.

## 1. Introduction

Since its origin in Wuhan, China in December 2019 [1], the novel coronavirus 2019-nCoV, or simply COVID-19, has quickly grown into a global pandemic, exposing the world to unprecedented health and economic challenges. As of 2 July 2020, the virus is believed to have infected more than 10.8 million people and contributed to over 520,000 of deaths worldwide. Severity of the pandemic has urged individuals to change their normal behaviors and governments to take unparalleled actions (travel restrictions, school and business closures, stay-at-home orders, among others), which have triggered a global economic downturn [2,3] and financial market turmoil [4,5,6]. Understanding the economic and financial market consequences of individuals’ and investors’ behaviors in the face of a pandemic represents an important consideration, both for policy design and financial strategy and planning.

In this study, we focus on one particular behavioral attribute of individuals under a pandemic: information search or, equivalently, risk perception. An essential aspect of the novel coronavirus is that upon its discovery it exposed individuals to a great uncertainty about the virus and its potential health and economic ramifications. It is human nature to seek out information about an unknown, which effectively amounts to risk perception, to inform subsequent decisions and actions. The present situation with COVID-19 is no exception. To understand the financial market implications of individuals’ behavior upon such uncertainty, we investigate the relationship between Google search queries related to the coronavirus—information search—and the performance of major financial indices. Using daily data between 22 January 2020 and 2 July 2020, we employ the Bayesian inference of a structural vector autoregressive (SVAR) model, where the endogenous variables are the percentage change in daily global Google search queries related to COVID-19 and the percentage change in daily financial market index. We consider a number of financial market indices, both from developed and developing countries, including S&P 500, Dow Jones, NASDAQ, FTSE 100, DAX, CAC 40, Nikkei 225, SZSE, and BSE SENSEX. Given that COVID-19 represents an exogenous shock, the percentage change in daily global COVID-19 confirmed cases (and deaths) is introduced to this framework as an exogenous variable, similar to Yilmazkuday [3,6] and Ahundjanov et al. [7].

**Figure 1:**
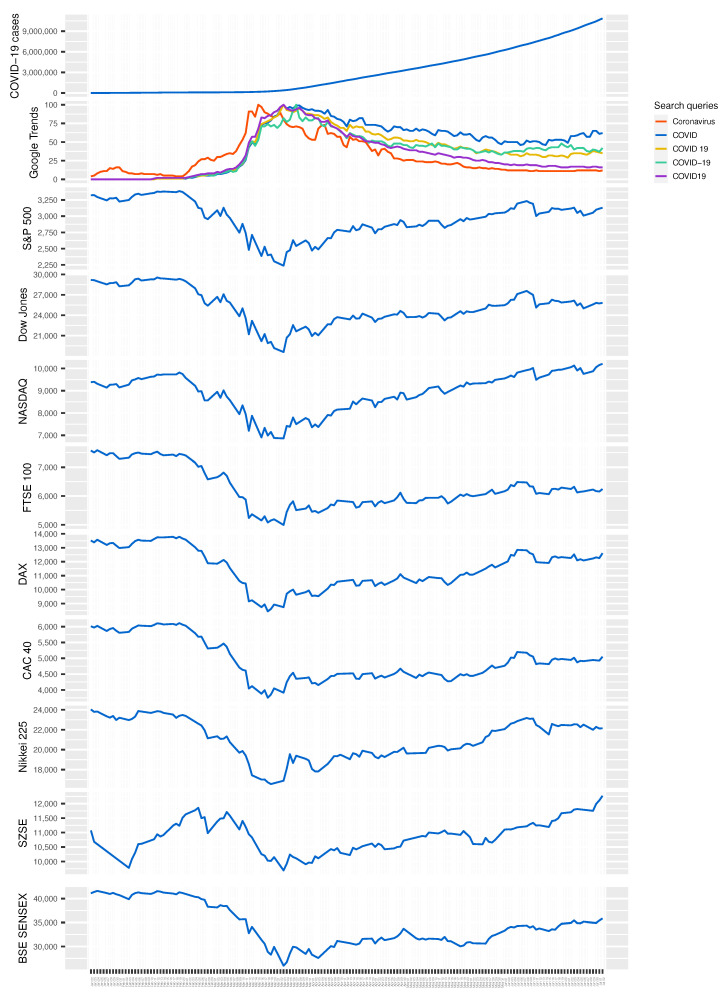
Time series plots of study variables between 22 January 2020, and 2 July 2020. Google Trends show the popularity of global search queries containing the term “Coronavirus”, “COVID”, “COVID 19”, “COVID-19”, or “COVID19”, which are commonly used to refer to the novel coronavirus. Google Trends data here shows the absolute trends for each of the five search queries; Figure 2 shows the relative trends for the five queries. A value of 100 is the peak popularity for a search query, while a value of 0 is the lowest popularity.

The strategy to use Google search queries to capture information search, attention, or, equivalently, risk perception, is consistent with prior literature and concurrent work on COVID-19 [8,9]. In particular, Google Trends, which provides data on Google search queries, has recently been used in finance [10], financial economics [11], monetary economics [12,13], and labor economics [14,15], among others. Furthermore, Google Trends data have also been shown to help predict home sales, automotive sales, and tourism [16]. Ginsberg et al. [17] document strong correlation between influenza epidemics and individuals’ health-seeking behavior in the form of online search queries. In essence, COVID-19-related search queries serve as a proxy for the demand for information about the virus which reflects an individual’s level of concern about the subject. Thus, the higher the search share, the higher the perceived risk among the population. This potentially leads to more intense financial market activities due to hedging against perceived risk. As Tett [18] noted it, in light of growing importance of online search activities, individuals and investors can manage and track investment returns with growing precision by plugging into online platforms. The time series plots of global Google search queries related to COVID-19 and financial market indices (see Figure 1) provide anecdotal evidence in support of this hypothesis: search queries (especially, those containing the term “Coronavirus”) and financial market indices are largely a mirror image of each other about the time axis.

Other approaches proposed in the literature to measure investor attention/risk attitude include market-based indices (e.g., trading volume, turnover, extreme returns), survey-based indices, and news-based indices (i.e., keywords or firm coverage in news media). Measuring attention/risk attitude based on aggregate search frequency in Google was popularized by the seminal work of Da et al. [19], which improves upon the other approaches in terms of capturing investor attention. Specifically, as Da et al. [19] noted, “aggregate search frequency in Google is a *direct* and unambiguous measure of attention” which allows for “capturing investor attention in a more timely fashion”.

The subsequent, and growing body of, studies have examined the effects of investor attention as approximated by aggregate search frequency in Google on asset prices and volatility [20] and investor behavior [10], among others. For a detailed review of this literature, see Kostopoulos et al. [10]. An important distinction between prior studies and our work is the type of Google search queries analyzed. In particular, previous literature focused on search queries related to individual stocks, such as company name and ticker symbol [19], or the economy, such as “financial crisis”, “recession”, “bankruptcy”, “deficit”, “unemployment”, and “inflation” [10]. In contrast, we focus on pandemic-related search queries, which represent a fundamentally different type of concern with its own effect on investor’s risk perception. Therefore, the efforts undertaken in this study contribute to our understanding of the implications of COVID-19-related information search on the performance of financial markets during the pandemic. We do not attempt, nor claim, to establish any causal relationship between the two variables. In  that sense, our study is similar to recent literature exploring associations between information search and investor behavior (see, for instance [10]).

**Figure 2:**
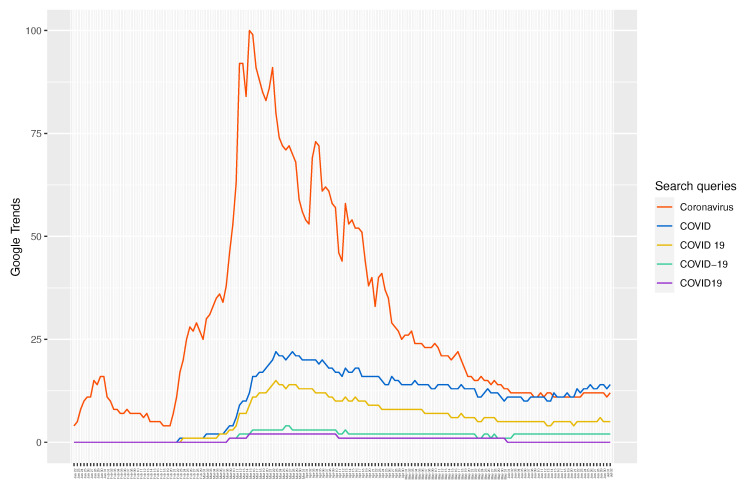
Relative trends of five Google search queries between 22 January 2020, and 2 July 2020. Google Trends shows the popularity of global search queries containing the term “Coronavirus”, “COVID”, “COVID 19”, “COVID-19”, or “COVID19”, which are commonly used to refer to the novel coronavirus. A value of 100 is the peak popularity for a search query, while a value of 0 is the lowest popularity.

The formal empirical analyses suggest that one unit increase (shock) in the popularity of Google search query “Coronavirus”, which is found to be the most popular query among COVID-19-related queries analyzed in this study (see Figure 2), is associated with 0.041%, 0.044%, 0.045%, 0.052%, 0.062%, 0.058%, 0.030%, and 0.036% of a cumulative decrease in S&P 500, Dow Jones, NASDAQ, FTSE 100, DAX, CAC 40, Nikkei 225, and BSE SENSEX indices, respectively, after one day and 0.069%, 0.073%, 0.074%, 0.085%, 0.104%, 0.099%, and 0.061% of a cumulative decrease in S&P 500, Dow Jones, NASDAQ, FTSE 100, DAX, CAC 40, and Nikkei 225 indices, respectively, after one week. More importantly, one unit increase in the combined popularity of all Google search queries related to COVID-19 (i.e., the sum of all COVID-19-related queries analyzed in this study) is associated with 0.054%, 0.056%, 0.058%, 0.057%, 0.069%, 0.065%, and 0.039% of a cumulative decrease in S&P 500, Dow Jones, NASDAQ, FTSE 100, DAX, CAC 40, and Nikkei 225 indices, respectively, after one day, and 0.104%, 0.114%, 0.108%, 0.118%, 0.150%, 0.143%, 0.095%, 0.054%, and 0.082% of a cumulative decline in S&P 500, Dow Jones, NASDAQ, FTSE 100, DAX, CAC 40, Nikkei 225, SZSE, and BSE SENSEX indices, respectively, after one week. As is evident, the effects for total search queries are more pronounced than those for a single query, which emphasizes focusing on aggregate Google search queries as opposed to a single query for sound empirical analysis and inferences. According to our findings, the Frankfurt Stock Exchange (DAX) index was relatively more sensitive to Google search queries related to COVID-19 than other indices analyzed in this study. The results are robust to a range of alternative specifications, including the inclusion of COVID-19 related deaths and a measure of global economic activity.

Generally, our study lies at the intersection of nascent literature on behavioral aspects of COVID-19 [8] and financial market implications of the pandemic [4,5,6]. The endeavor undertaken here bridges these two lines of literature, furthering our understanding of financial market consequences of individuals’ risk perception under a pandemic, which is essential for policy design and financial strategy and planning. As such, our study contributes to a broader literature exploring the effects of risk perception on economic choice [10,21].

The remainder of the paper is organized as follows. In Section 2, we review the concurrent studies on financial market implications of online searches. Section 3 describes the data used in this study, while Section 4 presents the empirical framework. The results and discussions appear in Section 5 and concluding remarks are in Section 6.

## 2. Related Literature

The literature in this area is nascent, but rapidly evolving. Toda [5], building and calibrating a stylized production-based asset pricing model, predicts that the stock prices temporarily decrease by about 50% during the epidemic, but recover quickly after the epidemic. Baker et al. [4] provide a descriptive comparison of the stock market volatility during all major infectious disease outbreaks, and argue that the impact of COVID-19 on the stock market is unparalleled. Our paper is related to a concurrent work by Yilmazkuday [6], who explores the impact of global COVID-19 related deaths on the S&P 500 index. Unlike this study, our paper focuses on a particular link between the COVID-19 and financial market performance (while controlling for COVID-19 cases): information search or risk perception. For comparison, Yilmazkuday [6] finds that a unit increase in global COVID-19 related deaths results in 0.02% of a cumulative reduction in the S&P 500 index after one day and 0.06% of a cumulative reduction after one week. In contrast, our analyses demonstrate that a unit increase in global search interest of COVID-19 results in 0.054% of a cumulative decline in the S&P 500 index after one day and 0.104% of a cumulative decline after one week. We show that the correlation of financial market indices is orders of magnitude stronger with Google search queries related to COVID-19 than with COVID-19 confirmed cases (or COVID-19 related deaths). Therefore, our study contributes to this line of work by providing a more complete picture of the implications of COVID-19 on financial markets in both developed and developing countries.

Papadamou et al. [22] also investigate the impact of Google Trends on major stock markets. (This literature was brought to our attention during the review process. We were not aware of it at the time when we completed this project. For full disclosure, the working paper version of our study was completed in mid-April and posted in *COVID-19 Projects* of the European Economic Association (EEA) in early May (https://www.eeassoc.org/index.php?site=JEEA&page=298&trsz=299), which seems to coincide with the publication date (4 May 2020) of the study by Papadamou et al. [22]). There are a number of differences between this study and our work presented here. First, and foremost, Papadamou et al. [22] focus on implied volatility index (VIX), while we focus on overall stock market indices. Both approaches have their own merits and have been used in the literature. Second, Papadamou et al. [22] collect search queries containing the term “corona virus”. In contrast, we  consider a range of terms related to COVID-19 (such as “coronavirus”, “COVID”, “COVID-19”, etc.) to capture various possible ways one can specify a search query in Google. As we showed above, the effect of aggregate COVID-19-related search queries (i.e., the sum of search queries related to COVID-19) is considerably larger than that for a single query (“Coronavirus”). Thus, the magnitude of estimates reported in Papadamou et al. [22] are likely affected by the ignorance of other possible ways to specify a COVID-19 related search query. Finally, the empirical approach taken by Papadamou et al. [22] is a panel VAR model, whereas we employ the Bayesian inference of a SVAR model.

Similarly, Amstad et al. [23] also examine the effect of investors’ risk attitudes as approximated by internet searches on global stock markets. (This literature was also brought to our attention during the review process. We were not aware of it at the time when we completed this project. For full disclosure, the working paper version of our study was completed in mid-April and posted in *COVID-19 Projects* of the European Economic Association (EEA) in early May (https://www.eeassoc.org/index.php?site=JEEA&page=298&trsz=299), which precedes the publication date (26 June 2020) of the study by Amstad et al. [23].) Again, there are several key differences between this study and our work. First, Amstad et al. [23] focus on stock market prices and equity returns, whilst we focus on overall financial market indices. Second, Amstad et al. [23] consider search queries containing the terms “coronavirus” and “COVID-19”, which is an improvement over Papadamou et al. [22], but it still leaves out several other (more popular) keywords. From Figure 2, it is clear that queries containing the terms “COVID” and “COVID 19” were more popular than those containing the term “COVID-19”. Consequently, this affects the total volume of internet searches considered in Amstad et al. [23], and thus the magnitude of estimates reported there. Third, the study period in Amstad et al. [23] begins from mid-February, whereas in our case it starts from mid-January. From  Figure 1, it is apparent that the Shenzen Stock Exchange (SZSE) index started to exhibit clear signs of disturbance in January, which can be explained by anxiety due to early cases of COVID-19 cases in China. This is also reflected in Google Trends, which shows a noticeable spike in the search interest of COVID-19 during this period. In fact, a close inspection of Google Trends (search query “Coronavirus”) and S&P 500 index between 22 January and 10 February reveals that the Western markets actually started to react to the then-unknown virus much earlier. Last but not least, Amstad et al. [23] implement an ordinary least squares (OLS) estimation, while we employ the Bayesian inference of a SVAR model, where impulse response function allows us to identify a shock in Google Trends and examine its dynamic effect on financial markets over time.

Taken together, the above studies complement and enrich each other, and combined produce a more rigorous empirical analysis offering perspectives from different lens.

## 3. Data

We construct daily data consisting of three data sets for the period between 22 January 2020 (which is the start date of the COVID-19 data set) and 2 July 2020: (i) global COVID-19 confirmed cases; (ii) COVID-19 related worldwide Google search queries; and (iii) major financial market indices. The coronavirus data are sourced from the Johns Hopkins University Center for Systems Science and Engineering (JHU CSSE). The data are publicly available at https://github.com/CSSEGISandData/COVID-19.

Coronavirus related online search queries are collected from Google Trends. The data are publicly available at https://trends.google.com/trends. We collected data for global search queries containing one of the five common terms used to refer to the novel coronavirus: “Coronavirus”, “COVID”, “COVID 19”, “COVID-19”, and “COVID19”. Search queries are not case sensitive. Google Trends data, which range from 0 to 100, represents search interest relative to the highest point on the chart for the given region and time. Thus, a value of 100 is the peak popularity for a search query, while a value of 0 is the lowest popularity. Figure 2 shows the *relative* trends of the above five queries. It is apparent that “Coronavirus” has been the single most popular search query during the study period. Thus, it is our first information search variable of interest. We also sum the data for five queries to get the total (combined) search volume over the study period, and use it as our second (main) information search variable. Although it is rather obvious to perform the analysis with total search volume, granted it reflects the total interest in the subject, we also perform the analysis with search data for “Coronavirus”, an approach taken by some literature [22], to show the difference in estimation results.

To study the heterogeneous impact of COVID-19-related internet searches on financial markets, we consider major financial markets in both developed and developing countries. Financial market data includes daily data (at market close) of S&P 500, Dow Jones Industrial Average, and NASDAQ Composite indices for the US; FTSE 100 index for the UK; DAX index for Germany; CAC 40 index for France; Nikkei 225 index for Japan; SZSE index for China; and BSE SENSEX index for India. The data for S&P 500, Dow Jones Industrial Average, and NASDAQ Composite indices come from FRED, Federal Reserve Bank of St. Louis. The data are publicly available at https://fred.stlouisfed.org. The data for other indices come from Yahoo!Finance. The data are publicly available at https://finance.yahoo.com. For consistency with other data sets, the missing observations in financial data (due to weekends and holidays) are linearly interpolated. For robustness, we also considered imputation by spline interpolation and Kalman smoothing [24]. Our main results, which are available upon request, were qualitatively unaffected. We also tried to run the analysis by aggregating COVID-19 cases and Google search data over the weekends and holidays. Again, our main findings were virtually unaltered.

**Figure A1:**
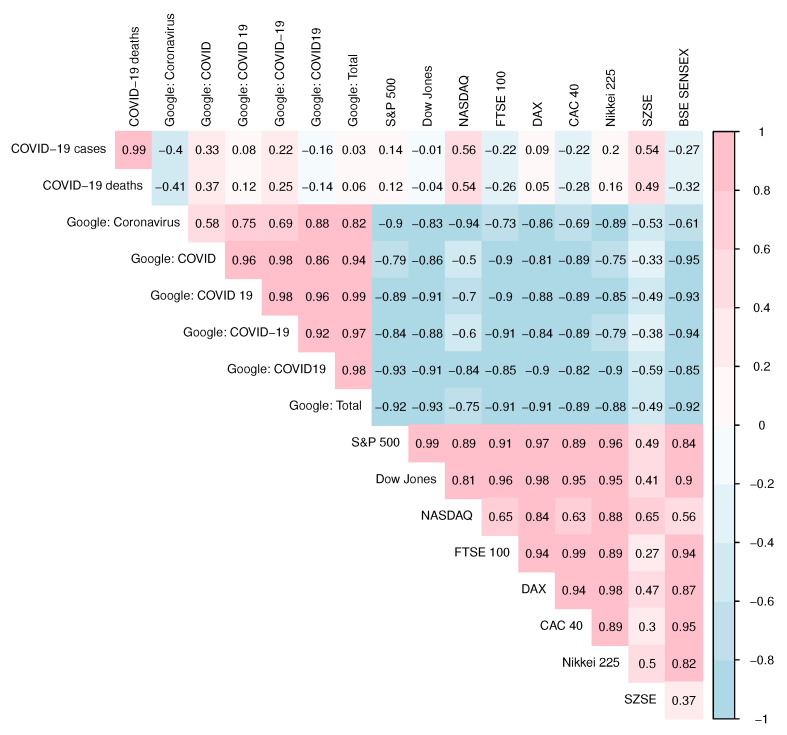
Correlation matrix for study variables. Pearson pairwise correlation coefficients are reported. ‘Google: Coronavirus’ is search query containing the term “Coronavirus” and so on, while ‘Google: Total’ is the sum of five search queries: “Coronavirus”, “COVID”, “COVID 19”, “COVID-19”, and “COVID19”. We include COVID-19 related deaths to show the strength of the correlation between this variable and financial market indices, and contrast that with the correlation between Google search queries and financial market indices.

The summary statistics for the study variables appear in Table 1. Figure 1 presents the time series plots of the study variables. With a rise in the novel coronavirus confirmed cases, the interest—information search—for it also gradually grew, with queries containing “Coronavirus” peaking on 15 March 2020. It is clear that the evolution of the major financial market indices over the study period is largely the mirror image that of Google search queries, particularly that of “Coronavius”, about the time axis. This anecdotally shows the reaction of individuals and investors (upon learning about COVID-19) to the pandemic in terms of their financial market decisions. Appendix A Figure A1 provides further evidence in this regard.

Interestingly, what Figure A1 in the Appendix A also reveals is that the majority of financial market indices are not as strongly correlated with COVID-19 confirmed cases (or COVID-19 related deaths) as one would expect. In fact, the correlation between financial market indices and Google search queries related to COVID-19 is orders of magnitude stronger. This, in and of itself, underscores the importance of information search (risk perception) to explain financial market implications of the pandemic.

## 4. Methodology

We implement a structural vector autoregressive (SVAR) model to study the relationship between individuals’ online search behaviors and financial market performance during a pandemic. An advantage of a SVAR model in this context is that it allows for identifying shocks and trace them over time by employing impulse response function. This explains a widespread use of SVAR model in the emerging COVID-19 literature [3,6,7,9]. Consider a bivariate vector of endogenous variables $y_{t}={\left(\Delta g_{t},\Delta f_{t}\right)}^{\prime }$ in period *t*, where $\Delta g_{t}$ is the percentage change in daily global Google search queries related to the COVID-19 (in our case, “Coronavirus” or the sum of five queries discussed in Section 3); and $\Delta f_{t}$ is the percentage change in daily financial market index (in our case, S&P 500, Dow Jones, NASDAQ, FTSE 100, DAX, CAC 40, Nikkei 225, SZSE, or BSE SENSEX). Similar to Yilmazkuday [3,6], we include percentage change in daily global COVID-19 confirmed cases $\Delta c_{t}$ as an exogenous variable, as it is not influenced by any economic variables. The Augmented Dickey–Fuller test for the study variables (i.e., $\Delta g_{t}$, $\Delta f_{t}$, $\Delta c_{t}$) strongly rejects the null hypothesis of a unit root, as shown in Table 1 (last column).

The SVAR model is specified as:

(1) $$\begin{array}{c} Ay_{t}=a+\sum_{k=1}^{p}A_{k}^{*}y_{t-k}+\Phi \Delta c_{t}+u_{t} \end{array}$$

where $u_{t}$ is the vector of mutually and serially uncorrelated structural errors (innovations). The optimal lag order *p* is determined based on Schwarz criterion from the candidate models containing 1–15 number of lags of $y_{t}$. For robustness, the lag order is determined using two alternative packages in R [25]: tsDyn (version 10-1.2) and vars (version 1.5-3) packages [26]. Both methods produced the same optimal lag orders for the models analyzed in the paper. It is conventional to express Equation (1) for estimation purposes in reduced form as follows:

(2) $$\begin{array}{ccc} y_{t} & = & A^{-1}a+\sum_{k=1}^{p}A^{-1}A_{k}^{*}y_{t-k}+A^{-1}\Phi \Delta c_{t}+A^{-1}u_{t}\\ & = & b+\sum_{k=1}^{p}B_{k}^{*}y_{t-k}+\Omega \Delta c_{t}+e_{t} \end{array}$$

where $A^{-1}$ is the structural impact multiplier matrix with a recursive structure such that the reduced form errors $e_{t}$ can be decomposed as $e_{t}=A^{-1}u_{t}$. The size of the shocks is standardized to unity, so that the identification is by triangular factorization [27]. The recursive structure of $A^{-1}$ inherently necessitates an ordering of endogenous variables in $y_{t}$ to allow a proper transmission of the shocks. We order the variables as $y_{t}={\left(\Delta g_{t},\Delta f_{t}\right)}^{\prime }$, which implies that shocks in $\Delta g_{t}$ (i.e., a Google COVID-19-related search query) can affect $\Delta f_{t}$ (i.e., a financial market index), which is the main goal of the present study. In order to allow shocks from $\Delta f_{t}$ to also affect $\Delta g_{t}$, thereby allowing the interaction between the two variables, we do not impose any block exogeneity. For robustness, we also estimated the model by imposing block exogeneity so that shocks in $\Delta f_{t}$ do not influence $\Delta g_{t}$ contemporaneously. The results, which are available upon request, were largely similar.

The SVAR model in Equation (2) is estimated by Bayesian inference [28], using the bvartools (version 0.0.2) package in R [25]. We use independent Wishart priors and perform a total of 10,000 iterations of the Gibbs sampler to obtain the posterior draws, of which the first 5000 is dropped as burn-in draws. The remaining 5000 is used for inferences and to construct a structural impulse response function (IRF). The IRF allows for investigating the dynamic interactions between the endogenous variables by illustrating the simulated impact (shock) of a unit change in variable *j* to the variable *i* at time *s*, for s=1,2,⋯ We employ a cumulative IRF to study the cumulative impulse response of the financial market indices to a unit change (shock) in Google search queries related to COVID-19.

## 5. Results

**Figure 3:**
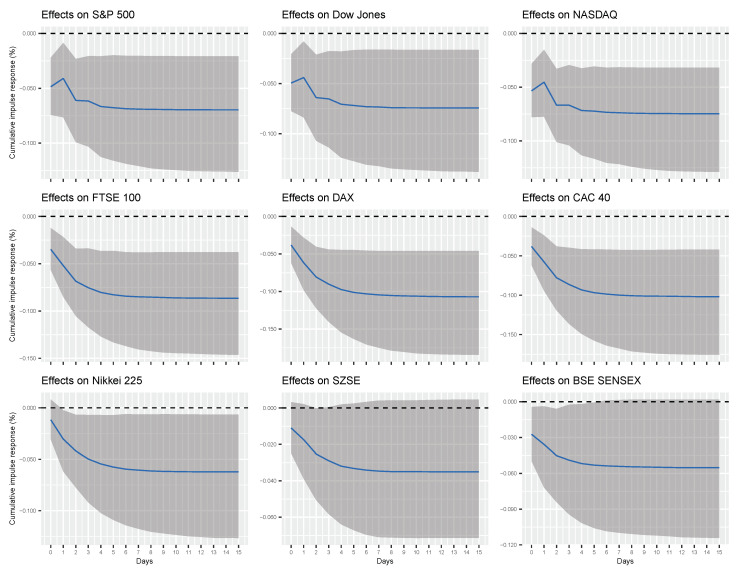
Cumulative effects of Google search query “Coronavirus” on financial market indices. The solid lines correspond to the Bayesian estimates, while the shaded regions represent 95% credible intervals.

**Figure 4:**
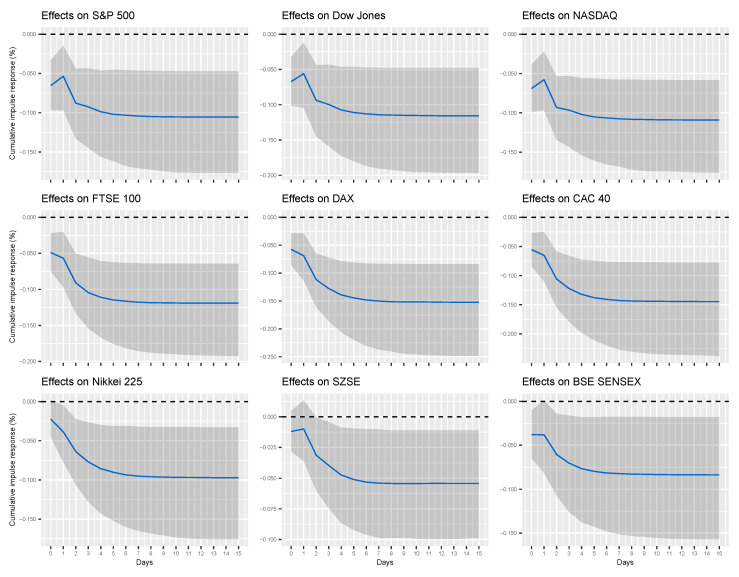
Cumulative effects of total Google search queries related to COVID-19 on financial market indices. The solid lines correspond to the Bayesian estimates, while the shaded regions represent 95% credible intervals. Total Google search queries is the sum of five search queries: “Coronavirus”, “COVID”, “COVID 19”, “COVID-19”, and “COVID19”.

Table 2 reports the cumulative impulse responses of financial market indices to a unit change (shock) in two alternative Google search variables (“Coronavirus” or the sum of five COVID-19 related search queries) for different time horizons (1 day and 1 week). Figure 3 and Figure 4, on the other hand, provide a fuller picture by showing the evolution of the cumulative impulse responses over time.

According to our findings, one unit increase (shock) in the popularity of Google search query “Coronavirus” is associated with 0.041%, 0.044%, 0.045%, 0.052%, 0.062%, 0.058%, 0.030%, and 0.036% of a cumulative decline in S&P 500, Dow Jones, NASDAQ, FTSE 100, DAX, CAC 40, Nikkei 225, and BSE SENSEX indices, respectively, after one day and 0.069%, 0.073%, 0.074%, 0.085%, 0.104%, 0.099%, and 0.061% of a cumulative decline in S&P 500, Dow Jones, NASDAQ, FTSE 100, DAX, CAC 40, and Nikkei 225 indices, respectively, after one week. From Figure 3, it is clear that these effects converge to their long-run levels in about one week. On the other hand, one unit increase (shock) in combined popularity of all Google search queries related to the COVID-19 (i.e., the sum of queries “Coronavirus”, “COVID”, “COVID 19”, “COVID-19”, and "COVID19") is associated with 0.054%, 0.056%, 0.058%, 0.057%, 0.069%, 0.065%, and 0.039% of a cumulative decrease in S&P 500, Dow Jones, NASDAQ, FTSE 100, DAX, CAC 40, and Nikkei 225 indices, respectively, after one day, and 0.104%, 0.114%, 0.108%, 0.118%, 0.150%, 0.143%, 0.095%, 0.054%, and 0.082% of a cumulative decline in S&P 500, Dow Jones, NASDAQ, FTSE 100, DAX, CAC 40, Nikkei 225, SZSE, and BSE SENSEX indices, respectively, after one week. Again, these effects converge to their long-run levels in about one week, as shown in Figure 4.

Several observations are worth mentioning. First, our findings show that the cumulative responses of financial markets are generally higher for changes in *total* volume of internet searches related to COVID-19 than for changes in search volume of a single query (i.e., “Coronavirus”). Although this observation is unsurprising, granted total volume of search queries subsumes “Coronavirus”, it is not yet reflected in concurrent work on COVID-19. Second, the effect of internet searches on financial markets is heterogeneous. In particular, (total) Google search queries have the highest cumulative effect on the Frankfurt stock exchange index DAX (−0.069 after one day and −0.150 after week), whereas the lowest effect on the Tokyo stock exchange index Nikkei 225 (−0.039 after one day) and the Shenzen stock exchange index SZSE (−0.054 after one week). On the other hand, the US and British financial markets tend to exhibit similar effects (about −0.056 after one day and −0.110 after week).

**Figure A2:**
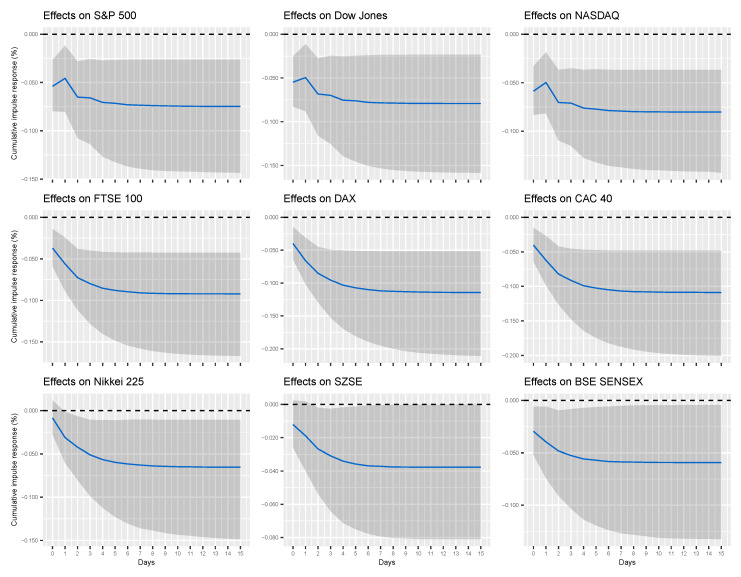
Cumulative effects of Google search query “Coronavirus” on financial market indices (with $c_{t-1}$ as an additional exogenous variable). The solid lines correspond to the Bayesian estimates, while the shaded regions represent 95% credible intervals.

**Figure A3:**
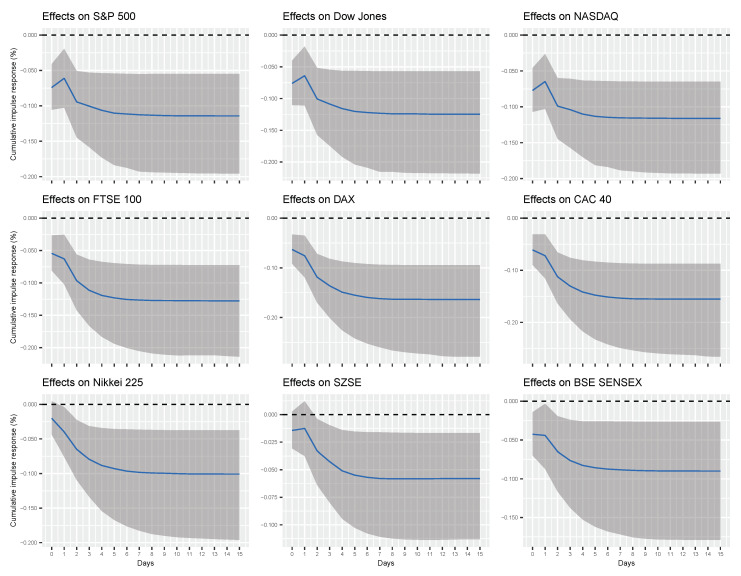
Cumulative effects of total Google search queries related to COVID-19 on financial market indices (with $c_{t-1}$ as an additional exogenous variable). The solid lines correspond to the Bayesian estimates, while the shaded regions represent 95% credible intervals. Total Google search queries is the sum of five search queries: “Coronavirus”, “COVID”, “COVID 19”, “COVID-19”, and “COVID19”.

**Figure A4:**
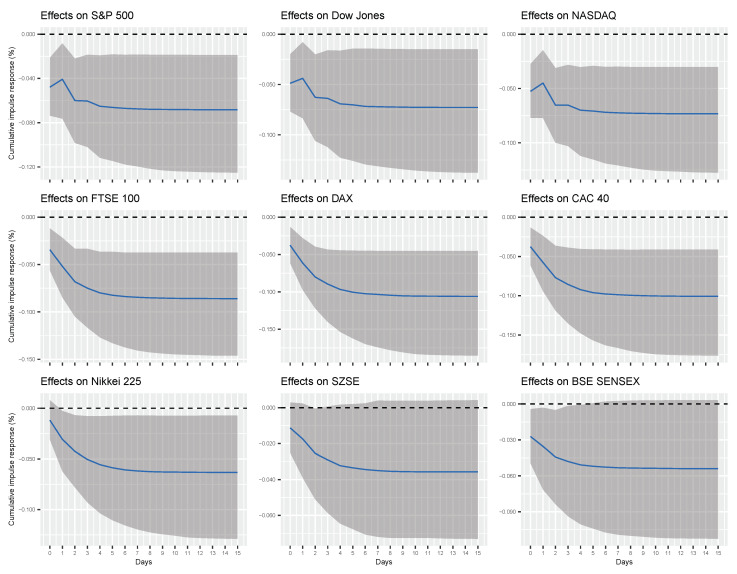
Cumulative effects of Google search query "Coronavirus" on financial market indices (with COVID-19-related deaths $d_{t}$ as an exogenous variable). The solid lines correspond to the Bayesian estimates, while the shaded regions represent 95% credible intervals. Total Google search queries is the sum of five search queries: “Coronavirus”, “COVID”, “COVID 19”, “COVID-19”, and “COVID19”.

**Figure A5:**
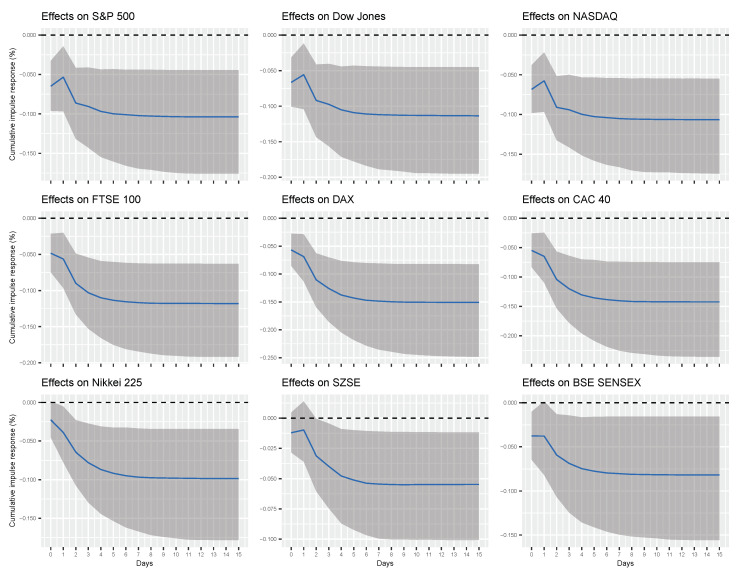
Cumulative effects of total Google search queries related to COVID-19 on financial market indices (with COVID-19-related deaths $d_{t}$ as an exogenous variable). The solid lines correspond to the Bayesian estimates, while the shaded regions represent 95% credible intervals. Total Google search queries is the sum of five search queries: “Coronavirus”, “COVID”, “COVID 19”, “COVID-19”, and “COVID19”.

**Figure A6:**
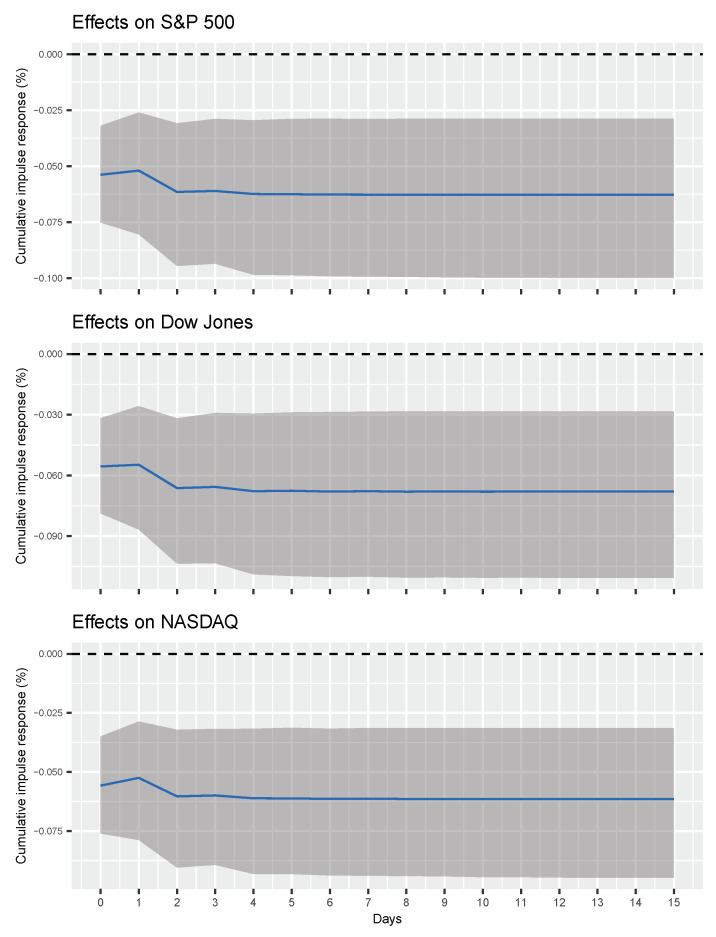
Cumulative effects of US-based Google search query “Coronavirus” on US financial market indices. The solid lines correspond to the Bayesian estimates, while the shaded regions represent 95% credible intervals.

**Figure A7:**
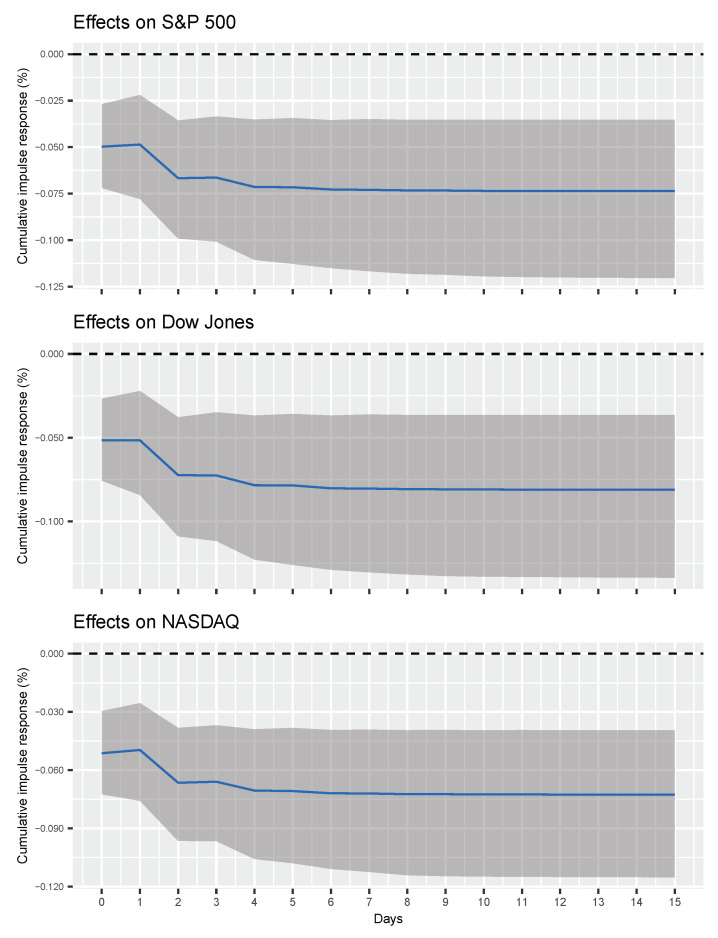
Cumulative effects of total US-based Google search queries related to COVID-19 on US financial market indices. The solid lines correspond to the Bayesian estimates, while the shaded regions represent 95% credible intervals. Total Google search queries is the sum of five search queries: “Coronavirus”, “COVID”, “COVID 19”, “COVID-19”, and “COVID19”.

To guard against the confounding effects of other factors on our findings, we performed a battery of robustness checks. First, to control for the possible lagged effects of COVID-19 confirmed cases, we estimated Equation (2) with $\Delta c_{t-1}$ as an additional control (see Figure A2 and Figure A3 in the Appendix A). Second, given that COVID-19 related deaths can potentially lead to a greater level of concern than COVID-19 confirmed cases per se, we also estimated Equation (2) using COVID-19 related deaths ($\Delta d_{t}$) as an exogenous variable (see Figure A4 and Figure A5 in the Appendix A). Third, given that financial markets are tightly intertwined with global economic activities, we also added the global economic activity measured by the Baltic Exchange Dry Index (BDI) as an additional endogenous variable in Equation (2). The BDI, published daily by the Baltic Exchange in London, effectively represents the changes in the global real activity and has been used extensively in the recent literature [29,30,31]. Fourth, for US financial markets, we also performed analysis with US-based Google search data, instead of global Google search data (see Figure A6 and Figure A7 in the Appendix A). Our main findings remain robust to all of these alternative specifications.

Our results are generally consistent with those of Yilmazkuday [6], who explores the impact of COVID-19 related deaths on the S&P 500 index. Given that financial market indices are correlated more strongly with Google search activities—information search—than with COVID-19 related deaths (see Figure A1), the estimates reported in Yilmazkuday [6] are orders of magnitude smaller than the ones reported here. Therefore, our study contributes to this emerging line of literature by providing a more complete picture of the implications of the COVID-19 pandemic on financial markets.

## 6. Conclusions

The novel coronavirus has spread to every corner of the world within a short period of time, crippling economic and financial markets in unprecedented manner. This study has focused on the financial market implications of online information search under such uncertain times. Using daily data between 22 January 2020 and 2 July 2020, in conjunction with a structural vector autoregressive model, we have investigated the relationship between Google search queries related to the COVID-19 and the performance of major financial market indices in developed and developing countries. The empirical analysis suggests that a one unit increase in the popularity of global Google search queries related to the COVID-19 results in 0.038–0.069% of a cumulative decline in global financial indices after one day and 0.054–0.150% of a cumulative decrease after one week. Various alternative specifications attest to the robustness of our findings.

The endeavor undertaken in this study makes couple distinct contributions to the literature with potential policy and empirical implications. First, and foremost, the analysis presented here furthers our understanding of the nature and magnitude of financial market consequences of individuals’ behavior—information search or, equivalently, risk perception—under a pandemic, which is critical for policy design and financial strategy and planning. Pandemics expose individuals, investors, firms, and policy makers to a great uncertainty about how they are going to unfold: individual health risks as well as economic and employment prospects during and in the aftermath of a pandemic. Such uncertainty contributes to a varying degree to an individual stakeholder’s risk perception, which is a key psychological factor guiding one’s economic choice [10,21]. Our study contributes to this literature by shedding light on how risk perception under a pandemic influences individual’s choice in terms of their financial market decisions. Second, our use of Google information search to capture risk perception during a pandemic is one of a kind. With increasing importance of online search and social platforms, publicly available data from platforms such as Google, Twitter, and Facebook proves indispensable in empirical research as it allows the analyst to tap micro-level data to form economic and psychological variables, such as preference and risk perception that enable the analyst to study various economic implications of these variables during uncertain times, such as during a pandemic [8]. This is clearly the growing trend in the literature [10,31], with fruitful applications in economics and finance.

## Figures and Tables

**Table 1:** Summary statistics for study variables.

Variable	Mean	Median	St. Dev.	Min	Max	ADF	ADF
						(for Levels)	(for % Change)
COVID-19 cases	2,984,018.51	1,845,226.00	3,234,202.74	555.00	10,871,926.00	0.675	−12.835 ***
Google: Coronavirus	29.83	20.00	24.91	4.00	100.00	−1.674	−4.958 ***
Google: Total	184.36	167.00	131.92	4.00	462.00	−1.781	−5.096 ***
S&P 500	2962.20	2978.76	274.37	2237.40	3386.15	−1.424	−5.599 ***
DJ	25,167.96	25,128.17	2616.20	18,591.93	29,551.42	−1.503	−5.613 ***
NASDAQ	8914.89	9150.94	857.90	6860.67	10,207.63	−1.271	−5.101 ***
FTSE 100	6259.09	6078.20	703.16	4993.90	7586.00	−1.327	−4.565 ***
DAX	11,506.96	11,541.87	1414.28	8441.71	13,789.00	−1.294	−4.149 ***
CAC 40	4911.80	4717.89	652.01	3754.84	6111.24	−1.253	−4.072 ***
Nikkei 225	20,977.48	21,082.73	1999.06	16,552.83	24,031.35	−1.529	−4.707 ***
SZSE	10,843.92	10,845.40	564.31	9691.53	12,269.49	−1.627	−4.771 ***
BSE SENSEX	34,305.88	33,412.83	4318.10	25,981.24	41,613.19	−0.934	−4.195 ***

*Note:* ‘Google: Coronavirus’ is search query containing the term “Coronavirus” and so on, while ‘Google: Total’ is the sum of five search queries: “Coronavirus”, “COVID”, “COVID 19”, “COVID-19”, and “COVID19”. The Augmented Dickey–Fuller (ADF) test is performed for variables in levels (second to last column) as well as variables transformed to daily percentage changes (last column). The null hypothesis for the ADF test is a series has a unit root. Significance of the ADF test statistics are indicated as follows: *** *p* < 0.01, ** *p*< 0.05, * *p*< 0.10.

**Table 2:** Cumulative effects of Google search queries on financial market indices.

	After 1 Day	After 1 Week
**Effects of Google search query “Coronavirus” on:**		
S&P 500	−0.041	−0.069
	[−0.076,−0.008]	[−0.121,−0.020]
Dow Jones	−0.044	−0.073
	[−0.084,−0.008]	[−0.132,−0.016]
NASDAQ	−0.045	−0.074
	[−0.077,−0.015]	[−0.122,−0.031]
FTSE 100	−0.052	−0.085
	[−0.085,−0.022]	[−0.141,−0.038]
DAX	−0.062	−0.104
	[−0.098,−0.028]	[−0.175,−0.046]
CAC 40	−0.058	−0.099
	[−0.094,−0.024]	[−0.168,−0.042]
Nikkei 225	−0.030	−0.061
	[−0.062,−0.002]	[−0.118,−0.006]
SZSE	−0.017	−0.035
	[−0.039,0.002]	[−0.071,0.004]
BSE SENSEX	−0.036	−0.054
	[−0.072,−0.004]	[−0.110,0.001]
**Effects of total Google search queries related to COVID-19 on:**		
S&P 500	−0.054	−0.104
	[−0.097,−0.014]	[−0.171,−0.046]
Dow Jones	−0.056	−0.114
	[−0.104,−0.012]	[−0.190,−0.048]
NASDAQ	−0.058	−0.108
	[−0.097,−0.022]	[−0.168,−0.058]
FTSE 100	−0.057	−0.118
	[−0.097,−0.020]	[−0.186,−0.064]
DAX	−0.069	−0.150
	[−0.113,−0.029]	[−0.237,−0.083]
CAC 40	−0.065	−0.143
	[−0.110,−0.025]	[−0.227,−0.077]
Nikkei 225	−0.039	−0.095
	[−0.077,−0.005]	[−0.165,−0.032]
SZSE	−0.010	−0.054
	[−0.036,0.013]	[−0.099,−0.010]
BSE SENSEX	−0.038	−0.082
	[−0.082,0.001]	[−0.151,−0.018]

*Note:* Reported are the Bayesian estimates and 95% credible intervals (in brackets). Total Google search queries is the sum of five search queries: “Coronavirus”, “COVID”, “COVID 19”, “COVID-19”, and “COVID19”.
